# Supplementary figures and images for: Severe fluctuation in mean perfusion pressure is associated with increased risk of in-hospital mortality in critically ill patients with central venous pressure monitoring: A retrospective observational study
Source: PLoS One. 2023 Jun 13;18(6):e0287046. doi: 10.1371/journal.pone.0287046 (PMC10263335; doi:10.1371/journal.pone.0287046)

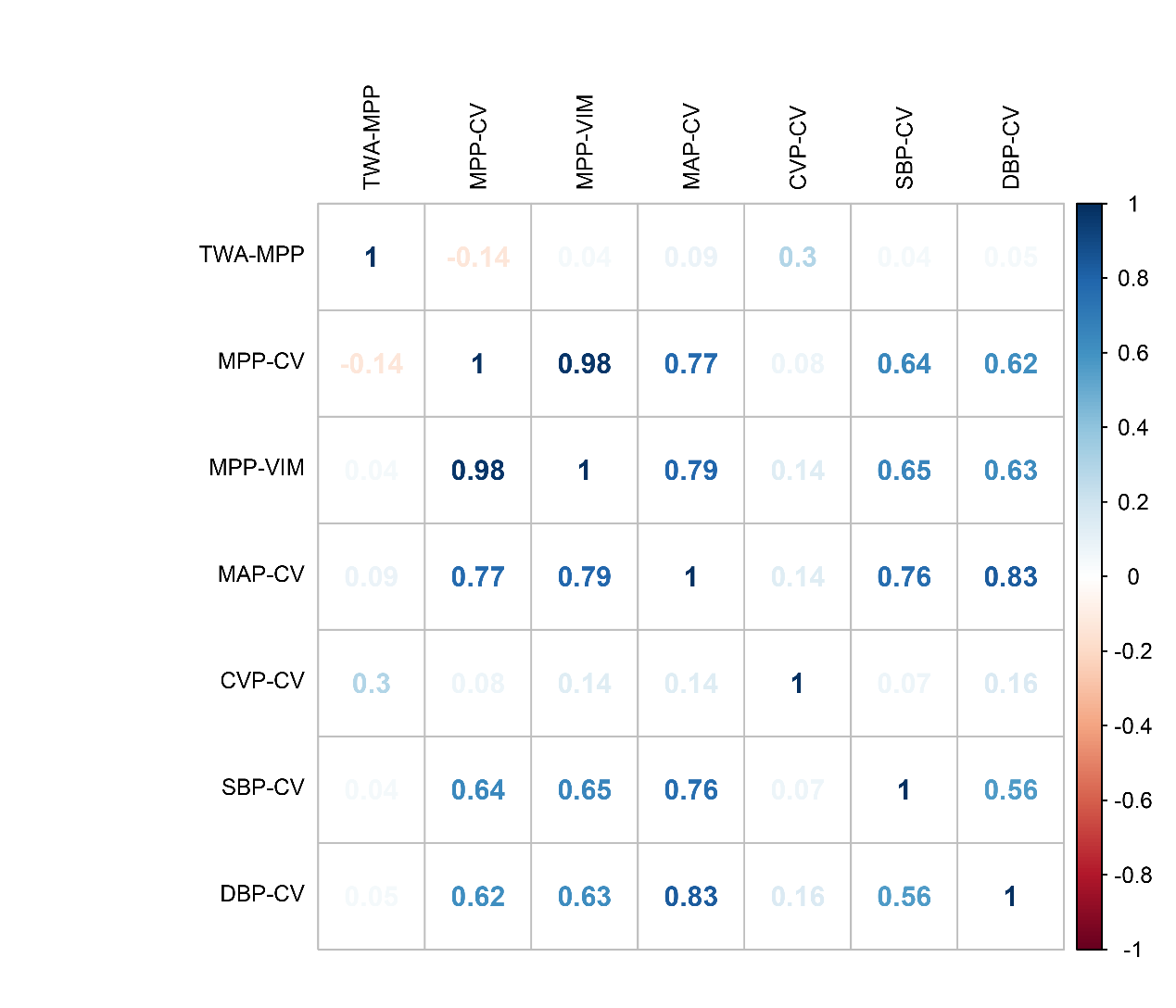

Supplement: S1 Fig — (TIF) [file pone.0287046.s001.tif]

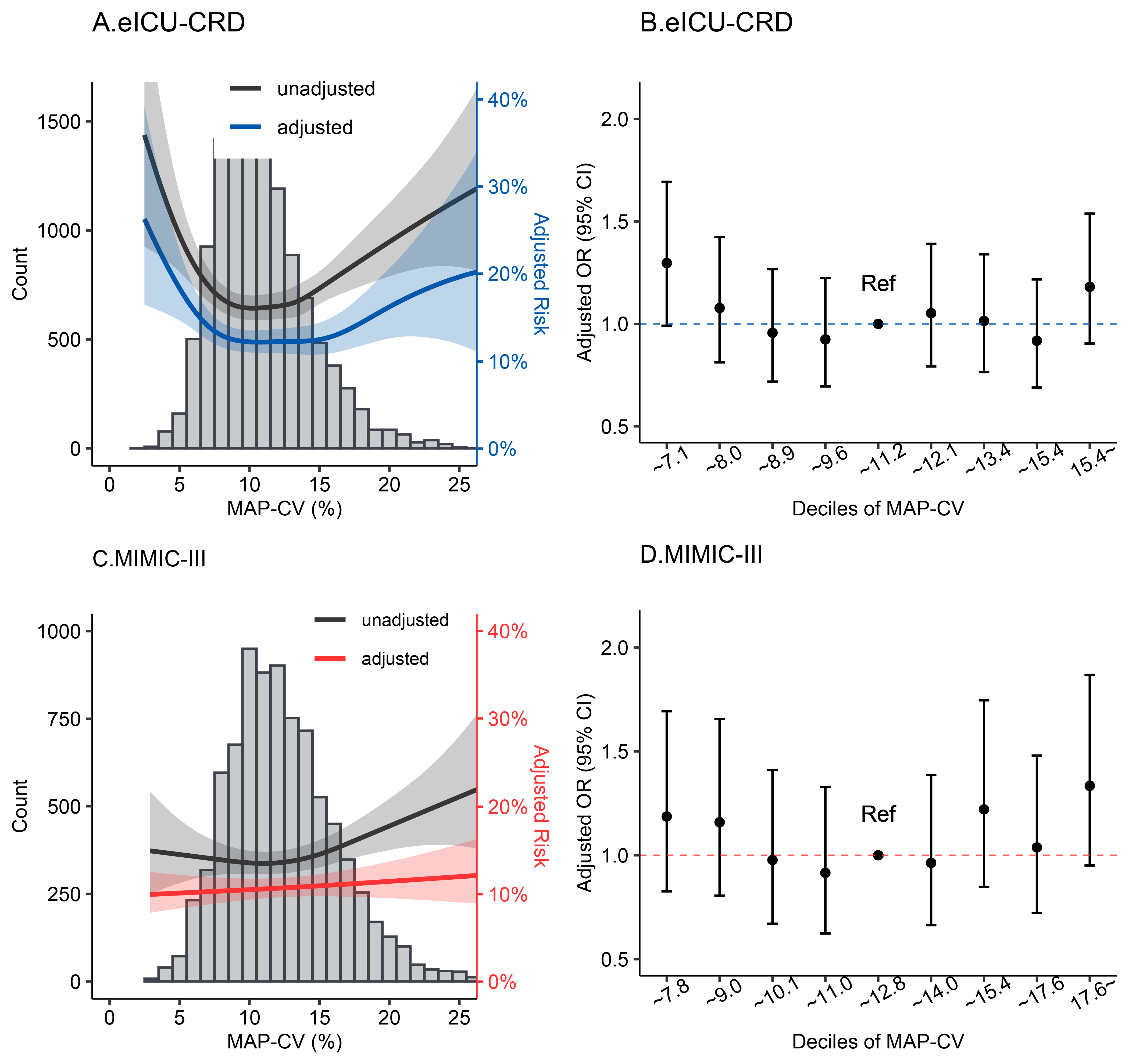

Supplement: S2 Fig — (TIF) [file pone.0287046.s002.tif]

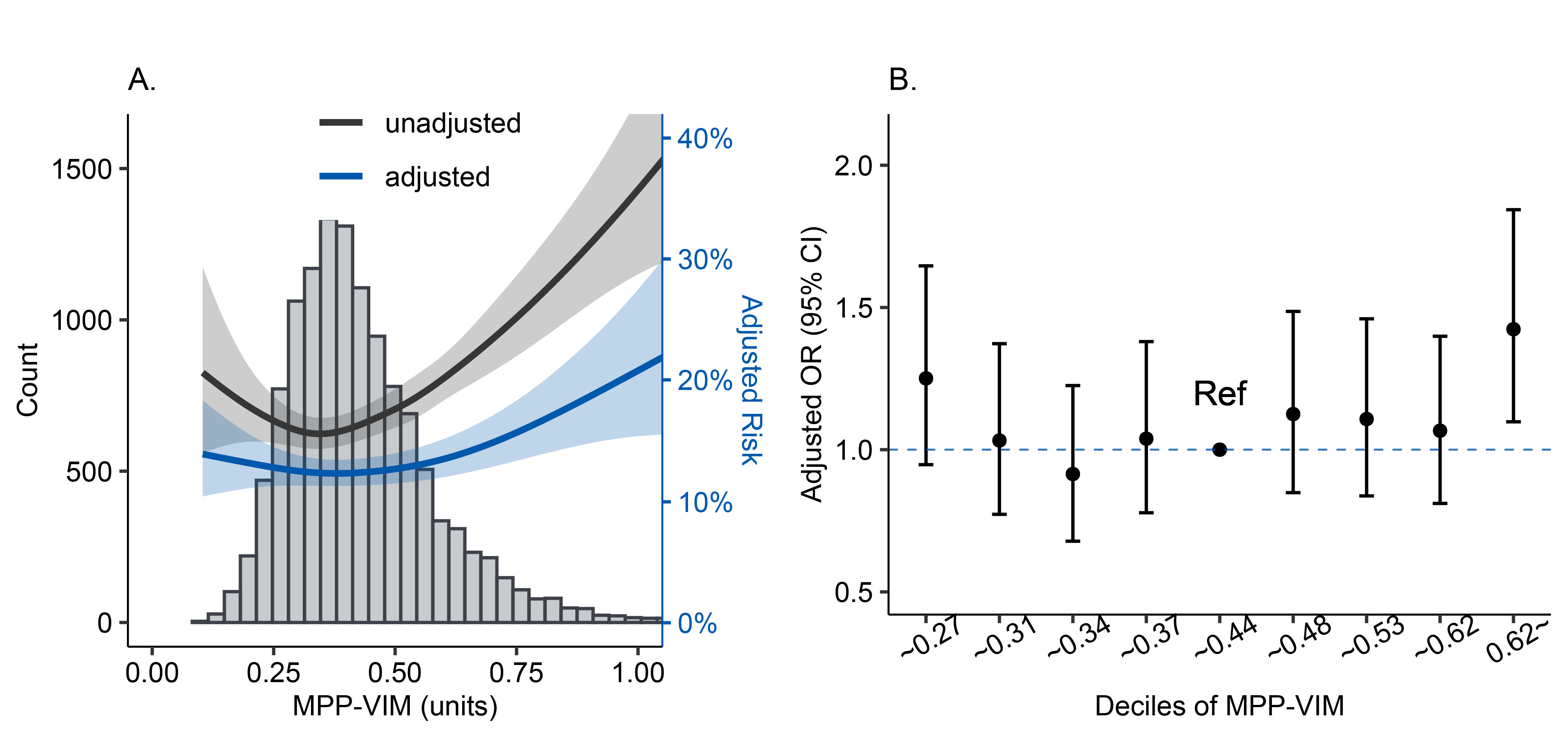

Supplement: S3 Fig — (TIF) [file pone.0287046.s003.tif]

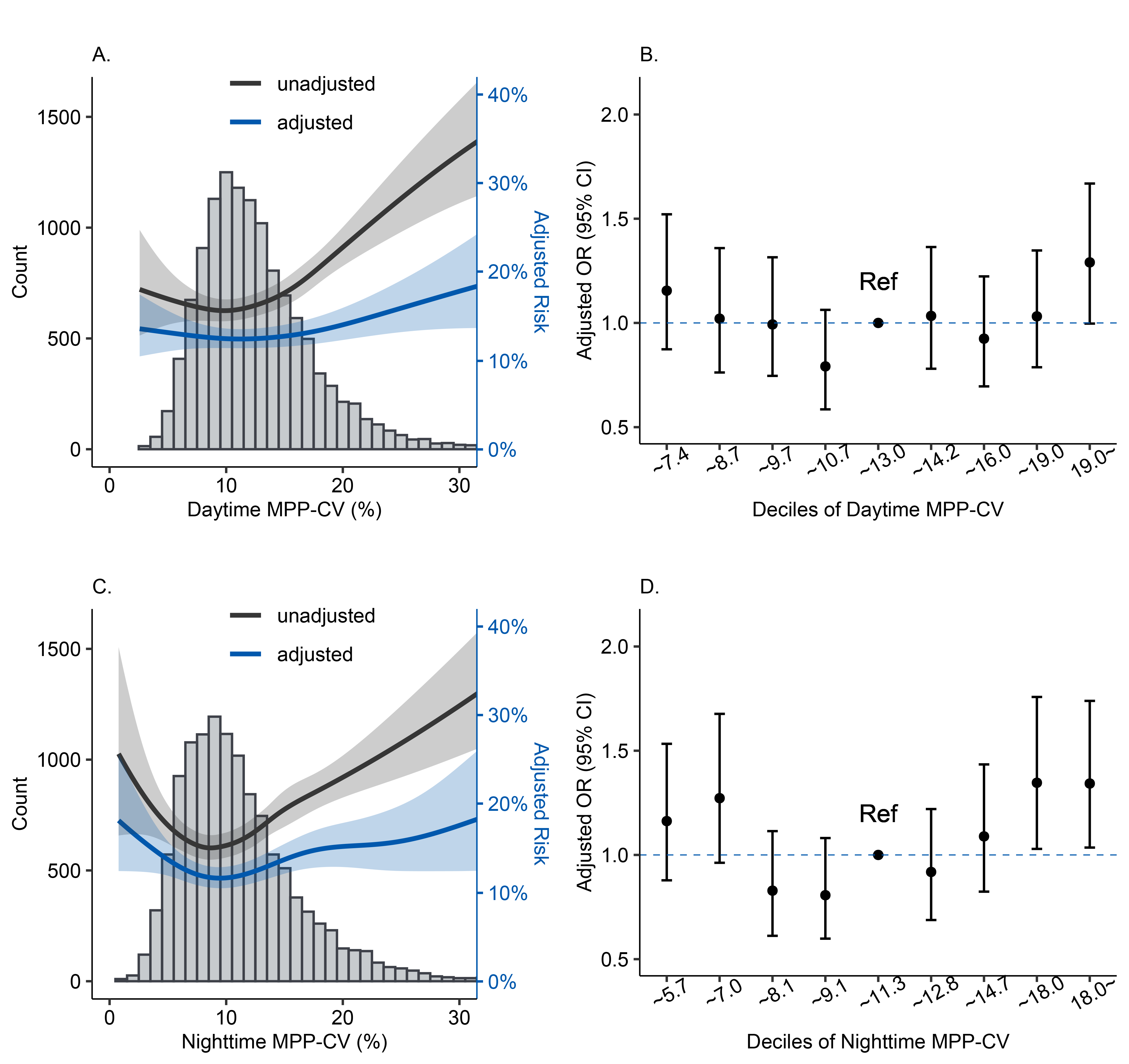

Supplement: S4 Fig — (TIF) [file pone.0287046.s004.tif]

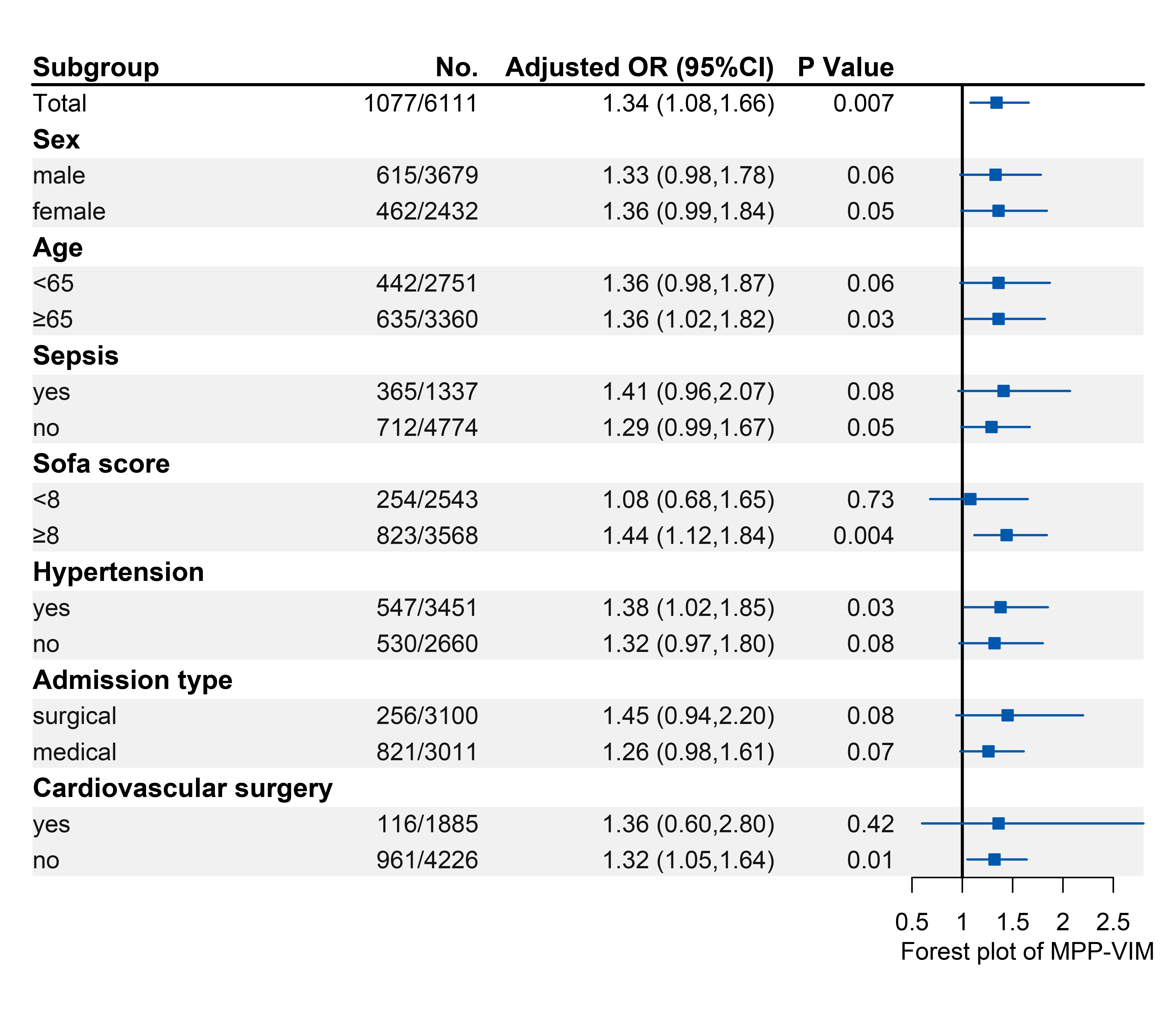

Supplement: S5 Fig — (TIF) [file pone.0287046.s005.tif]

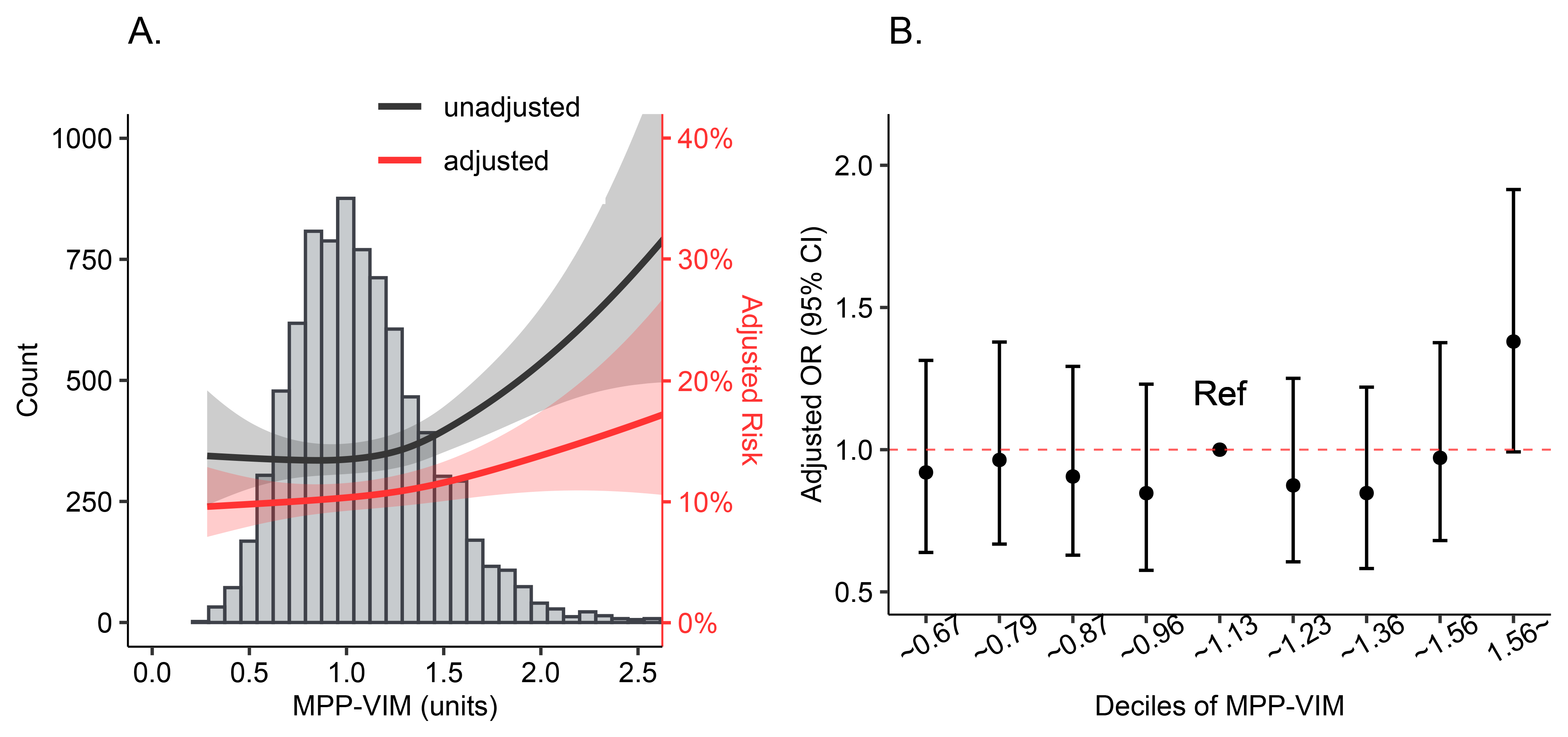

Supplement: S6 Fig — (TIF) [file pone.0287046.s006.tif]
