# Supplementary material for: Severe fluctuation in mean perfusion pressure is associated with increased risk of in-hospital mortality in critically ill patients with central venous pressure monitoring: A retrospective observational study
Source: PLoS One. 2023 Jun 13;18(6):e0287046. doi: 10.1371/journal.pone.0287046 (PMC10263335; doi:10.1371/journal.pone.0287046)
Supplement: S1 Table — Note: n is the number of MPP readings, x¯ is the mean value and w refers to the time of each interval. For VIM, linear regression fitting log (SD) with log (x) was performed. The “k” was the exponential of β0 and the “b” was the β1 of the linear regression model. (DOCX) [file pone.0287046.s007.docx]

**Supplementary Table 1. Calculation Formula of variability parameters.**

| **Parameter** | **Formula** |
| --- | --- |
| Coefficient of variation  (CV) | $\frac{SD}{\bar{x}}$ |
| Variation independent of mean  (VIM) | k$\times\frac{SD}{\bar{\bar{x}^{b}}}$ |

Note: n is the number of MPP readings, $\bar{x}$is the mean value and w refers to the time of each interval. For VIM, linear regression fitting log (SD) with log (x) was performed. The “k” was the exponential of β0 and the “b” was the β1 of the linear regression model.
