# Supplementary material for: Severe fluctuation in mean perfusion pressure is associated with increased risk of in-hospital mortality in critically ill patients with central venous pressure monitoring: A retrospective observational study
Source: PLoS One. 2023 Jun 13;18(6):e0287046. doi: 10.1371/journal.pone.0287046 (PMC10263335; doi:10.1371/journal.pone.0287046)
Supplement: S2 Table — Continuous variables were expressed as median (interquartile range) as the distributions are skewed and categorical variables were expressed as number (percentage). ICU: intensive care unit; MPP: mean perfusion pressure; TWA: time weighted-average. (DOCX) [file pone.0287046.s008.docx]

**Supplementary Table 2. Other information of the study population.**

| Variables | N=6111 |
| --- | --- |
| Hospital size |  |
| <100 | 63 (1.0) |
| 100 - 249 | 2933 (48.0) |
| 250 - 499 | 811 (13.3) |
| ≥ 500 | 1497 (24.5) |
| Not available | 807 (13.2) |
| Teaching hospital | 2488 (40.7) |
| Region (%) |  |
| Midwest | 2114 (34.6) |
| Northeast | 358 (5.9) |
| South | 1677 (27.4) |
| West | 1604 (26.2) |
| Not available  Admission type  Medicine  Elective surgery  Urgent surgery  Cardiovascular surgery | 3009 (49.2)  2762 (45.2)  338 (5.5)  1885 (30.8) |
| Initial diagnosis |  |
| Sepsis | 917 (15.0) |
| Cardiovascular | 3545 (58.0) |
| Respiratory | 441 (7.2) |
| Gastrointestinal | 531 (8.7) |
| Genitourinary | 81 (1.3) |
| Neurologic | 283 (4.6) |
| Others | 313 (5.2) |
| Comorbidities (%) |  |
| Hypertension | 3451 (56.5) |
| Diabetes | 1953 (32.0) |
| Chronic kidney disease | 828 (13.5) |
| Chronic heart failure | 1134 (18.6) |
| Malignancy | 91 (1.5) |
| MPP data |  |
| Measurement times | 283 (262,288) |
| First MPP measurement after ICU admission (minutes) | 103 (10, 565) |
| TWA-MPP (mmHg) | 63.0 (57.1, 69.9) |
| Mean MPP (mmHg) | 63.0 (57.1, 69.9) |
| Outcomes |  |
| In-hospital mortality (%) | 1077 (17.6) |
| ICU mortality (%) | 795 (13.0) |
| Hospital length of stay (days) | 10.8 (6.9, 18.0) |
| ICU length of stay (days) | 4.4 (2.8, 8.0) |

Continuous variables were expressed as median (interquartile range) as the distributions are skewed and categorical variables were expressed as number (percentage).

ICU: intensive care unit; MPP: mean perfusion pressure; TWA: time weighted-average.
