# Supplementary material for: Severe fluctuation in mean perfusion pressure is associated with increased risk of in-hospital mortality in critically ill patients with central venous pressure monitoring: A retrospective observational study
Source: PLoS One. 2023 Jun 13;18(6):e0287046. doi: 10.1371/journal.pone.0287046 (PMC10263335; doi:10.1371/journal.pone.0287046)
Supplement: S3 Table — AUC: area under the curve; CI: confidence interval; MPPV: mean perfusion pressure variability; MAPV: mean arterial pressure variability. (DOCX) [file pone.0287046.s009.docx]

**Supplementary Table 3. The comparison of the AUC between MPPV and MAPV in prediction the hospital mortality.**

|  | AUC of MPPV (95% CI) | AUC of MAPV (95% CI) |
| --- | --- | --- |
| CV | 0.56 (0.54-0.58) | 0.50 (0.48-0.52) |
| VIM | 0.54 (0.52-0.56) | 0.50 (0.48-0.52) |

AUC: area under the curve; CI: confidence interval; MPPV: mean perfusion pressure variability; MAPV: mean arterial pressure variability.
